# British Pathology

**Published:** 1822-06-01

**Authors:** 


### Medico-Chirurgical Review,

AND

### JOURNAL OF MEDICAL SCIENCE.

(Analytical Series.)

"Nec Aranearum textus ideò melior, quia ex se fila tingunt; nec noster vilior, quia ex alienis lubamus, ut apes."

Vol. III.] JUNE 1, 1822. [No. 9.

### I.

### BRITISH PATHOLOGY.

*Elements of Pathology and Therapeutics; being the Outlines of a Work intended to ascertain the Nature, Causes, and most efficacious Modes of Prevention and Cure of the greater Number of the Diseases incidental to the Human Frame; illustrated by numerous Cases and Dissections.* By Caleb Hillier Parry, M. D. F. R. S. Member of the College of Physicians of London; Physician to the General Hospital at Bath, &c. &c. Vol. I. General Pathology. Octavo, pp. 464. London, 1815.

Some of our readers may be surprized at our taking up a work that has been seven years before the public. It is true that it has been seven years before the profession, but they are not yet acquainted with it. We have reason to believe, or rather to know, that not *four* hundred copies have been distributed among a British professional public, of at least eight thousand individuals; consequently the work is virtually unknown to nineteen-twentieths of medical society. Were it a book of common merit, we should let it take its chance; but believing it to be one of the most enlightened productions that ever issued from the press, in any age or in any country, we should hold ourselves culpable if we did not give it that publicity which may insure it an attentive, as well as an extensive, perusal. In the first number of the *monthly* series of this Journal, we attempted this object; but our well-meant labour failed on account of the then limited circulation of our Journal. The case is altered. We can now make known—effectually known, the merits of a publication, from Delhi in the East, to Cincinnati in the West.

It is true that Dr. Parry's work has been *reviewed* in medical journals. But, alas! as one of the most able writers of the age remarks—" modern *criticism* too often discloses that which it would fain conceal, but conceals that which it professes to disclose—it is therefore read by the discerning, not to discover the merits of an author, but the *motives* of the critic."*

The course which we pursue cannot well come under the above censure; for it is obvious that our public duty is also our private interest—that of charging our pages with useful matter from others, rather than captious criticism from ourselves.

The work now before us, we confidently predict, will stand a monument of fame to the author (now, alas! insensible to flattery or censure) " ære perennius," and an honour to the age and country in which it saw the light.† With the exception of a few points of doctrine, we believe that these " Elements of Pathology" will defy the ordeal of criticism, and finally receive from the profession at large, a sentence the most honorable and gratifying which an author can wish, or the public voice confer.

It is a work, indeed, which may justly be characterized as " pregnant with thought, and matured by reflection ;" but it is, on this account, less suited for the lower than the higher classes of the profession. There is a terseness, a precision, and an aphoristic conciseness in every sentence, that will assuredly cause it to appear dry, or even obscure, to the sciolist, and to the less studious members of the medical world; but which must render the work more valuable to those who look beyond the surface of things, and study with care the mechanism, the functions, and the lesions of the human frame.

The extent of our analysis will, therefore, be proportionate to the value which we set upon the publication; and we cannot pay a greater compliment to the author, or confer a greater benefit on our readers, than by allotting a portion of our Review to the dissemination of Dr. Parry's labours,

---

* Reverend Mr. Coulton's Lacon.

† One great reason, indeed, for our here bringing forward an extended review of Dr. Parry's work, is to shew our own, and our continental brethren what enlightened views of pathology have been entertained in this country, founded, *not* solely on post mortem appearances, as is too often the case, but chiefly on an attentive observation of morbid phenomena in the living body. The world will see that many doctrines now making much noise on the continent and in this country, have been completely anticipated by our own illustrious pathologist seven years ago.

which will at once evince the estimation in which we hold them, by the reluctance with which we shall part from them.

The work is divided into one thousand and twenty-three aphorisms or passages, of unequal lengths, and apparently with little regard to order or arrangement. This *plan* we humbly conceive to be injudicious, inasmuch as it is very uninviting to the reader; but Dr. Parry, no doubt, trusted to the sterling merit of the *matter* without shewing any very fastidious regard to the *manner* in which it is conveyed.

After some observations on the nature of medical science, our author enters on a consideration of the disordered states of the sanguiferous system, which form the most obvious deviations from health, of any incidental to the animal frame. These consist in some excess or defect in the quantity or velocity of the circulation. The *quality* of the circulating mass is left out of consideration. The *general* velocity of the blood may be natural, while in particular *parts* of the body it may be retarded or accelerated. So also in regard to quantity; the excess or deficiency may be either general or local; that is, there may be excess in one part and deficiency in another, or there may be general excess or deficiency in every part of the system at the same time, *Excessive* quantity is denoted by quick motion of the heart; preternatural fulness of the arterial, capillary, and venous system, and vice versa. The habits of mankind in civilized society tend to produce excessive nutrition, and consequently plethora in the human frame. This state of the sanguiferous system characterizes nine-tenths of the diseases of persons in moderately affluent circumstances. Blood is the pabulum from which, as it circulates through the capillaries or elsewhere, caloric is sensibly evolved, by whatever power that evolution may be effected. An increase of momentum and quantum in the circulation of a part is characterized by preternatural redness and size of that part, and especially if the arteries leading to, and the veins receding from that part, be unusually distended. The *quantity* of blood in the whole system remaining the same, a diminution in one part must produce plethora in all, or some other parts of, the system, and vice versa. The same may be said of the *momentum* of the blood. The coats of arteries possess two powers of motion; the first is mechanical elasticity, the other a vital or muscular power, denominated by our author tonicity. It is something similar to muscular action, but our author denies that the middle coats of arteries are *fibrous*. They have, however, "an inherent capacity of motion," and that is enough. In the larger arteries the mechanical, in the capillaries, the *tonic* power prevails. Our author believes with

Haller, that the force of the heart *alone*, in health, is adequate to the task of carrying on the march of the blood through its entire circulation. He concludes, that muscular contraction in the vessels themselves, would just as much impede the ingress of a new quantity of blood, as promote the egress of that already existing in them. However it may be explained, it is known, that certain vessels become unusually distended with blood, not only from excessive impulse by the heart, but *without* that impulse, as where shame fills the cutaneous arteries of the face, neck, &c. One of the most important phenomena of the animal frame is, that after vessels have been more or less robbed of their blood, a contrary state succeeds, termed *reaction*, altogether inexplicable, but on the principles of vitality. From our author's observations on the structure and functions of the sanguiferous system, we shall only extract the following passage.

"The arteries and veins being in a state of health always full, and the blood being incompressible, that fluid may be considered as if it formed a continuous solid column all the way onwards from the mitral to the tricuspid valves. Hence, at every systole or contraction of the left ventricle of the heart, the shock acts at the same instant throughout the entire circle." 34.

Our author justly observes, that could we calculate, from the heart's action, respecting the proper quantity of blood in the whole, or any part of the system, great benefit would result; but the pulse is now as it was two thousand years ago—"res fallacissima." Thus, in many diseases, the pulse of the radial artery will be small, while that of many other tangible arteries will be full and strong. Again, the face and head will often be flushed and hot; the pulse of the carotids strong, full, and bounding; while all the extremities are cold and pale, and the pulsation of their arteries small and weak. In gout, and local inflammations without fever, the arteries leading to the diseased parts will throb violently, while other arteries are tranquil or below par. This remark ought to be kept in mind, as it would often lead to the detection of disease. A general, but not universal law of the human constitution is, that excessive morbid determination to two different parts shall not exist in the same person at the same time. This is exemplified not only in gout, but in several other diseases; for he who has a catarrh to-day, may have a fit of the gout to-morrow; then a discharge of blood from the hæmorrhoidal veins, and shortly afterwards, a rupture of the medullary substance of the brain, from sanguineous effusion. This change of determination is a most interesting subject in animal pathology, and has been long caught at in the treatment of diseases, though with not so much success as could be wished. Thus it is usually in vain that we attempt to solicit to the extremities that excessive determination of blood which is called gout, though we employ topical heat, friction, blistering, &c.

Our author is of opinion, that although a suspension or cure of a disease may, perhaps, be occasionally produced by certain violent causes, (as he had seen asthma suspended by a blow on the head) yet, in the common order of conversions, the new affection or determination is, in point of time, either co-existent with the disappearance of the old one, or more usually subsequent to it; so that in reality, we cannot cure a malady by bringing on the gout, but must first cure the malady, and then, in a predisposed constitution, there is a fair chance that gout will supervene. The same principle is applicable to a great number of other occasions of the highest practical importance, as will be hereafter shewn.

*Inflammation and its consequences.* After enumerating the various characteristics of inflammation, as it affects different tissues, our author remarks that, of that termination strictly called resolution, or return of the colour and size of parts to precisely their former state, without any discharge, he never met with a single instance. In all *these* terminations, our author asserts, and we are perfectly of his opinion, that some exhalation from the over-distended vessels takes place, either into the cellular substance of the part, or neighbourhood, or from the cutaneous surface itself externally, or membranes, as the pleura, peritonæum, &c. internally. Of the other terminations we need not speak, but the foregoing remark is to be borne in mind. When constitutional symptoms accompany local inflammation, if blood be drawn in a projecting stream from a vein in the arm, and received into *small vessels with polished internal surfaces,* and suffered to cool slowly, it will exhibit a coat of fibrine, or what is called the inflammatory buff. But if other portions of blood be taken from the same patient at the same blood-letting, into *larger vessels,* or into one with a rough inside, as queen's-ware, though they be first drawn, and having the freest flow, no such crust will be seen. This ought also to be borne in mind. Although this appearance affords a presumption of some local inflammation, it is not absolutely decisive. It occurs during pregnancy—in morbid determinations, as hæmorrhage, &c. and also at the commencement of synocha, before any local inflammation exists. It also is occasionally seen in the blood of very old persons, and in those of a *nervous* disposition, where there is total exemption at the time from topical inflammation.

Dr. Parry, by a long chain of reasoning, attempts to prove, contrary to the theory of Dr. Philip, that in all inflammations, there is not only dilatation and increased quantum of blood in the vessels of the part, but that there is an increased *velocity* or momentum of the vital fluid, particularly when to the *local* inflammation is added *general* fever. Of this increased velocity we much doubt, notwithstanding the arguments of our author; nor has he convinced us of the "influence of the systole of the left ventricle on the returning venous blood," because "it frequently happens, that in gouty inflammation of the wrist, blood taken from the cephalic vein is propelled *in jets*, as strong as those from the temporal artery, and precisely synchronous with the pulse in the radial artery in the other arm." 78. We think that this curious phænomenon (which we have witnessed in many instances where there was no gout or local inflammation at all) might be accounted for otherwise than by supposing that the systoles of the heart "extended their projectile force through the capillaries, and nearly back through the entire course of the circulation."* 79. Dr. Parry offers some ingenious and sensible observations on what is termed the "*proximate cause*" of a disease, defining it—"that phænomenon, in the body or part, most *immediately preceding* the state which we call disease, without which previous phænomenon, the disease is not known to exist." 91. In this point of view, an increased momentum in the velocity of the

---

* If a heart be taken from a living animal and put into warm water, it will continue to *dilate* as well as contract, for a considerable time. If the heart of a turtle, for instance, be held in the hand, the *force* of dilatation is just as great, or nearly so, as that of contraction. Why then should not the dilatation of the cardiac cavities act as decisively in *abstracting* blood from the *venous* system, as the contractions of these cavities in *propelling* it through the arterial? We see no reason to the contrary; indeed, we firmly believe that the heart acts in this double capacity; and that the great object of the trunks of vessels is to *convey* the blood to the capillary system, having the power of adapting their calibres to the ever varying current through them. The uses of the capillary vessels, we believe, to be very different from, and very much more important than, that of *propelling* the blood. They have *secretion* to perform in *every* part of the system—in the muscles as well as in the glands. They have to change the blood from its arterial to its venous state, whatever may be the nature of that change. These important offices, however, are not sufficient in the eyes of many physiologists. The capillaries must *circulate* the blood, although there is a most powerful and wonderful organ for that express purpose! *Rev.*

blood through a part, and also a dilatation of the vessels of that part, may be considered as the proximate cause of inflammation.

Speaking of the process by which Nature cures an inflammation, our author justly observes, that after increased action, the heart generally falls into a state of unusual quiescence; while, at the same time, the general mass of blood is more or less diminished: (for instance, by the diminution of increment during all inflammatory affections.) It is, therefore, difficult to say, whether an exhaustion of the heart, in consequence of over exertion, or an abstraction of a quantity of that blood which is so great a stimulus to its action, be, in this case, the chief cause of its quiescence: both probably concur. 94.—On the other hand, if, by undue stimulation, too sudden a restoration of the plethoric state succeeds, and the heart is again excited to excessive action, the local disease, or some vicarious one, is readily produced. This we have so frequently seen bring on relapses after febrile diseases, that we heartily concur with Dr. Parry, when he deplores the mischief that too often results,—

"When, under the delusive notion of strengthening the patient, he is pampered with full diet, and stimulated with every description of drachms, whether solid or fluid, which the elaboratory of the cook, the vintner, or the apothecary can supply." 96.

In fact, the quantity of blood, suited to the salutary performance of the circulation, is by no means always the same, even in the same person. Thus, a man in the prime of life and health shall habitually have a considerable fulness of the sanguiferous system, yet enjoy an exemption from disease.

"But should the same man, after having been emaciated, suddenly grow full of blood, some violent disease, or succession of diseases, will usually again occur, and reduce the habit to its former state of emaciation."

A multifarious series of this kind will sometimes occur, during convalescence from long and violent diseases; and, in other cases, where health and strength have succeeded to great extenuation, and have continued for months, and even years, no sooner has the habit, by degrees, recovered its prior fulness, than the same, or a similar acting disease, shall return, and reduce the patient to another emaciation. This course we see continually exemplified in gout, erysipelas, and various other diseases, which are called constitutional.

"And, indeed, they are so frequent," says Dr. P. "that one cannot help considering the increased action of the heart, and the general circumstances of the disease connected with it, as natural efforts to remove a degree of fulness incompatible with the due performance of the healthy functions of that individual constitution." P. 97.

Under circumstances of *previous reduction*, from whatever cause, what morbid effects do we not daily see, even from an improper meal—from a strong mental emotion—from watching—wine, &c. which, in health, would never be noticed? Is is not, that in such cases, the heart is more susceptible of the stimuli applied to it?

Dr. Parry next enumerates the various structures of the human body, according to the arrangement of Bichât, and while he admits that each tissue is subject to its own peculiar modifications of disease, *in general*, he yet remarks, that the disease of one tissue will sometimes spread to another; and doubts " whether, in *all* the different textures, there is not *one* component or constituent part which is primarily affected in that morbid change called inflammation." 100.

It has been before remarked, that all terminations of inflammation are attended with more or less extravasation, and the same so commonly occurs in cases of general increased impetus, as in sweating after strong exercise, exposure to heat, palpitation of the heart, fever, &c. in all of which, a reduction of the increased impetus follows the effusion, that we cannot help conceiving the evacuation to be a process expressly designed for the purpose of mitigating or curing the disease.

Wherever important functions are to be performed, there we find a large supply of blood, by means of arteries terminating in an infinite number of capillaries, and disposing those very parts particularly to congestion or inflammation. But the benevolent author of our existence has made ample provision against these occurrences. Thus these various parts or organs are abundantly furnished with exhalents or secretory vessels from their capillaries. First apparatus, a simple surface, as the skin and mucous membranes. Second, natural cavities, the internal surface of which is lined with similar membrane, as the stomach, bowels, bladder, &c. Third, discontinuity of substance, forming virtual, though not often real cavities, into which exhalents open, as the cellular system, ventricles of the brain, medulla oblongata, nerves, duplicatures of the pleura, peritonæum, pericardium, synovial receptacles, &c. Fourth, an excretory duct or ducts, as in the mammæ, liver, kidnies, &c. &c. Most of these organs have a combination of two of these circumstances of structure, so as to acquire a double power of evacuation. Thus the lungs have pleura without, and mucous membrane within. The liver, peritonæum without, and pori biliarii from within. To these may be added, the cellular, parenchymatous, and other substances, affording a third emunctory for the superfluous contents of blood-vessels, by means of exhalents and secretory capillaries every where opening into them. To these contrivances there are only two exceptions, the thyroid gland and spleen.

The extravasated fluids resulting from inflammation are various. The simplest form is that of serum effused into the cellular membrane, as in superficial gouty inflammation; it is a true dropsy or œdema of the part. The same occurs as a termination of various inflammations; as of the gums, in caries, &c. of the teeth; of the leg, in sciatica; of the joints, in rheumatism; of the lungs, in peripneumony; of the forehead, temples, and eyelids, in erysipelas; in all parts, from external injuries, as blisters, &c. The same kind of effusion is poured out on the surfaces of the serous membranes, as the pleura, pericardium, &c. It is not confined, however, entirely to serous membranes:

"For in certain cases of pulmonary hectic an expectoration often occurs of a dense, hard, globular substance, partly transparent, and partly of a pearly whiteness, which immediately falls to the bottom of the water into which it is thrown, and has much the appearance of thickened albumen." 107.

Dr. P. is of opinion that albumen and fibrine are increased by all inflammatory processes in the constitution. The deposition of fibrine in particular, Dr. P. thinks, may account for various painless, but hard and obstinate swellings, which succeed inflammations, especially about the tendons and joints. Our author believes, and we think with reason, that small parts of the body may be made to unite again, after having been entirely separated, as Dr. Balfour and Mr. Bailey have indeed lately proved.

Another termination of inflammation is the effusion of blood itself. We see this in the bloody spots or streaks in the sputa of bronchitic and pneumonic patients, which, when slight, are always favourable. In pulmonic and hepatic inflammation, however, blood is often *fatally* effused into the air-cells and pori biliarii. Even from the surfaces of serous membranes entire blood is sometimes exhaled, especially about the period of death, both previously and subsequently. That termination of inflammation denominated Pus is not, even to this day, exactly characterized, since it (pus) is hardly ever procured unmixed with other fluids. Dr. Parry is inclined to believe that real pus may be secreted from unbroken surfaces.

"In cases of hemiplegia," says he, "in which blood is effused into the medullary substance of the brain, that fluid may be seen in all the intermediate states from entire blood, through what in appearance exactly resembles pus, to complete absorption." 122.

Dr. P. next takes a review of the various morbid, or rather *salutary* effusions and secretions which result from inflammatory processes, or increased momentum of the blood; previously observing, "that on a great variety of occasions, the effect of inflammation is to increase the natural secretions of the parts affected." But we are of opinion that, "on a great variety of occasions," the *contrary* is the case, and particularly in one of those instances which he has brought forward in corroboration of his opinions, viz. hepatitis. We have had opportunities of seeing this disease on a much larger scale than Dr. Parry; and from very attentive observation, we believe we may say with safety, that, during inflammation of the liver, there is in reality a *paucity* of the biliary secretion. We know, indeed, that discharges of morbid bile sometimes take place during the inflammatory process, and are speedily ejected from their irritating qualities; but such occurrences are not to warrant the conclusions which our author has drawn. Our author is of opinion, and with reason, that although these discharges from inflamed vessels are sometimes fatal (as in hydrocephalus or pneumonia for instance,) "the process, in a great majority of cases, is beneficial to the animal frame." 128. Among the fluids effused from inflammation, Dr. P. enumerates that cream-like substance deposited in the cavities of joints, in capsular ligaments, in the sheaths of tendons, &c. which, by the absorption of the thinner parts, becomes what is called chalk-stone, a well-known effect of highly-inflammatory gout, and consisting of urat of soda. The same is deposited in the cellular space between the inner and fibrous coats of the larger arteries, becoming true bone, or phosphate of lime, and producing such distressing effects, resulting, according to our author's observations, from an inflammatory affection of the vasa vasorum; and when on the coronary arteries of the heart, predisposing to syncope angens. Nodosity of the joints, Dr. P. supposes to be owing to "an erroneous deposit of common bone."

It has been shewn, that the capillary arteries have a power of contracting to their natural size, after being preternaturally dilated with blood. This power, however, seems to diminish, *cæteris paribus*, in proportion to the violence and duration of the distending cause:

"Hence," says our author, "one can scarcely avoid considering the state which follows, as what Dr. Cullen would have called collapse, consequent on undue excitement; and Dr. John Brown, indirect debility. But whatever name may be given to this state, the order of facts is not the less true." 135.

Dropsy. One of the simplest and most common terminations of inflammation is extravasation of serum, or one of its constituents; thus the swelling, which often accompanies the cessation of gouty paroxysms, is a true dropsy of the anasarcous kind, following, in free spaces, the direction of gravitation: "where, however, it is considerably extensive, it seems to arise *against gravitation* relatively to the inflamed part." This phænomenon, Dr. P. thinks, may be explained by supposing that the expulsory power of the exhalents overcomes the force of gravity in a column confined all around. Since we find that in gout, œdema continues to extend itself in proportion as the inflammation in the foot subsides, and often exists long after all symptoms of local inflammation are gone, "we cannot help," says Dr. P. "attributing its occurrence, in the latter case, to such a state of momentum of blood still existing in the vessels leading to the part originally inflamed, as produces preternatural evacuation by exhalation." And, on the same principle, the anasarca or œdema, extending to a distance from the original *seat* of inflammation, "originates in a similar condition in the neighbouring arteries and exhalents; both of which appear, from all the phænomena in such cases, to be preternaturally distended with blood." 141. Thus, in œdematous swellings of a lower extremity, following gout in the foot, if we apply a bandage to the foot and ankle, we shall still find an œdematous swelling recur every night *above* the bandage, and often in a greater degree than before the bandage was applied. Hence we may conclude, that *increased momentum* is sufficient to produce anasarca or œdema, without the existence of local inflammation as the source from whence the effusion takes place. Dr. P. illustrates this reasoning by the œdematous swellings of lower extremities following scarlatina, and also the ascites which not unfrequently supervenes, both evidently originating in the high phlogistic diathesis then prevailing in the system.

The fluid effused in what are supposed to be idiopathic ascites, hydrothorax, and anasarca, has all the chemical qualities of common serum; and the identity of the fluid thus effused with that which arises from certain degrees of inflammation in the same membranes, is certainly a strong argument in favour of a state in them approaching to the phlogistic diathesis. 143. This idea is farther strengthened by the thickening and opacity of the peritonæum and pleura, unaccompanied with disease of the liver or other part, which we see in ascites and hydrothorax, and which would justify our inferring a previous topical inflammation in those membranes. This inflammation proceeds in so slow and chronic a manner, with little or no pain or fever, and with only some symptomatic irregularity in the alvine excretions, that the patient is scarcely aware of the existence of important disease till he is alarmed by a preternatural tumefaction of the belly. So, our author has seen hydrothorax slowly arise from a diseased state of the pleura following slight inflammation, accompanied with habitual fever and high-coloured urine, but without the smallest affection whatever of the organs of respiration, till after the lapse of several months, when, at the end of a few days, the patients died, and dissection exhibited copious serous extravasation, without any pulmonary disease. 145. Anasarca of the lower extremities is also often preceded in them by local pains, which do not go the length of inflammation, and subside as the effusion takes place. In such cases, we cannot reasonably expect to find the secreting membranes to be always in a morbid state; for here, as in acute inflammation, the exhalation is the cure, or at least the effect of the curative process of the excreting parts. Hence the explanation of Bichat's assertion, that in increased secretions, the vascular system supplying the part, is not generally found more full than on other occasions.

The fluid found in the cavities of the brain in hydrocephalus is not serum, or at least contains little of it. It is not coagulable by heat or acids, though a small proportion of albumen is precipitated from it by the voltaic pile. It is worthy of attention, that dropsy is often evidently produced, and when existing aggravated, by many of those circumstances which are known to increase the momentum of the blood, as intemperance gives rise to ascites and anasarca without hepatic disease. From all these circumstances, it is probable, that although serous effusions are usually the consequences of local inflammation of either cellular or serous parts, yet they may occasionally take place from a degree of excessive momentum short of that which would have been necessary to produce either of these two states. As inflammation rarely occurs till some time *after* increased momentum of blood, so extravasation uniformly obeys a similar law, as may be illustrated by the obvious example of sweating, which rarely supervenes till a considerable time after the increased action of the heart from heat, exercise, &c. On other occasions of increased momentum, as in inflammation, effusion is not always proportioned, either to the mere disposition in the capillaries, or to the degree of increased momentum; but is relative to the sum of the two taken together. 148.

"Hence it will be found, that in certain states of the capillary system, even the healthy impetus may be sufficient to cause effusion; while, in other states, very great degrees will not produce the same effect, either at all, or else only through the medium of local inflammation. Ib.

This principle, our author thinks, will comprehend all the more essential examples of idiopathic dropsy, as well those styled *active*, as those attributed to debility.

"In this view, the malady in question, and the state of inflammation, throw on each other reciprocal light; since it appears that both have in common the circumstance of a degree of momentum of blood, which is excessive with regard to the whole, or a part of the animal system in general, or of that particular animal, or accidental constitution of the animal, which is the subject of the malady." 149. "If these facts be well founded, general dropsy, like the extravasations of inflammation, is to be considered rather as a salutary effort of the constitution to diminish morbidly increased momentum, than as a primary or actual disease."

In this, as in the former case, the effusion, occurring in certain parts, as in the ventricles of the brain, may prove fatal. This principle also illustrates the efficient and final causes why dropsy of various kinds follows venous and glandular obstruction; the arterial blood, thus arrested in its natural course, pouring out its serum through the exhalent extremities of the containing vessels. The phlogistic diathesis, our author thinks, may not be essential to serous effusion, or dropsy; though few dropsies will be found to exist without the appearance of inflammatory crust in the blood, at some stage or other of the disease. We may also conceive that yielding state of vessels, approaching to that of death, which, in order to the production of effusion, does not require the coincidence of excessive momentum.

It ought always, however, to be kept in mind, that in all these cases, the impetus is excessive with regard to the individual constitution, and therefore is speedily followed by the process of effusion. Dr. Parry remarks, that diminished absorption, as a cause of dropsy, is utterly untenable, his observation not having furnished him with a single fact in support of the agency of this cause. We fully agree with him.

Hæmorrhage.—As hæmorrhage frequently attends discharges of mucus, pus, and serum from inflamed parts, as the bronchia, liver, &c. it is considered by our author, as the topical effect of that increased momentum, forming a part of local inflammation. The same effect may happen from general plethora, as is shown by the bloody urine following scarlatina, and sometimes measles; forming, in scarlatina, one link of a series of effects, of which articular inflammation and dropsy often constitute other links. Here anasarca or ascites being vicarious with hæmorrhage, affords new evidence of the nature of dropsy, as discussed on a former occasion. Our author has more than once seen obstinate and extensive anasarca cured by spontaneous hæmorrhage. Active hæmorrhage is always accompanied with phlogistic diathesis, and when the blood is discharged from the nose or viscera, it appears to be arterial, either from ruptured arteries or open mouthed capillaries.

Dr. Parry here mentions "a modification of purpura, exhibiting those appearances under the skin, which from their size and shape have been generally called petechiæ, maculæ, or vibices. They are usually flat, but sometimes considerably relieved above the rest of the skin. They are of different tints and shades of colour, from a pale red to a logwood purple, or even nearly the hue of a black currant. They do not become in any degree fainter from pressure, and are evidently ecchymoses or spots of extravasated blood. Their chief, but not only seat, is the upper or lower extremities. In most cases they have followed or accompanied pretty severe pain in the limbs, which has sometimes had the form of articular inflammation." P. 156.

Dr. Parry has published two cases in the 5th vol. of the Edinburgh Journal, and we ourselves have witnessed within these few weeks a similar disease, which embarrassed us much at the beginning.

A young gentleman, after a hearty supper of oysters, was seized next day with bilious vomitings, head-ache, and considerable pyrexia. The fever continued for several days, with great determination to the head, notwithstanding strong cathartics were daily administered. About the sixth or seventh day, he was seized with violent pain in the small of the back, and in the region of the kidnies; soon after which, an eruption, similar to that above described, came out on the lower extremities, accompanied with excruciating pain in the limbs, and great tenderness and irritability on the surface; fever and restlessness still continued; and the intestinal discharges were invariably dark coloured and offensive. For three days, he took a grain of calomel and two of antimonial powder every three or four hours, which kept up a constant catharsis, and finally carried off the complaint. The appearance of these petechiæ would, we presume, a few years back, or even among our Brunonian brethren of the present day, have given origin to a very copious exhibition of bark and wine, to correct the putrescency of the humours and the debility of the solids.

Hæmatemesis and hemiplegia are brought forward by our author; the latter he has seen rapidly follow a violent palpitation of the heart, and was occasioned by a large extravasation of blood into the medullary substance of the brain, under which the patient lingered several weeks. In these cases of hemiplegia, and in many other hæmorrhages, the patient is, to all appearance, in such a state of previous good health, that one can hardly suspect phlogistic diathesis, or attribute the accidents to any other cause "than simple excessive momentum, acting perhaps, on vessels previously disposed to be so affected." Hæmorrhages may arise from general excessive momentum alone, as an exciting cause. Thus globules of blood are sometimes seen mixed with the perspired fluid in the axillæ of young persons after violent exertions; strong exercise, late hours, close rooms, or exposure to the intense heat of fires or sun will produce nasal hæmorrhage, and the same is produced by hard drinking in older persons.

"Sanguinis a naso fluxum, fere lethalem, Juveni a coitu prima nuptiarum nocte sæpius repetito accidisse novi."

In respect to passive hæmorrhages, Dr. Parry seems a little puzzled, as there is seldom any indication of increased momentum.

"But if," says he, "a great degree of momentum be required to produce hæmorrhage in vessels little disposed, a slight degree will be sufficient in vessels which are strongly disposed. These, therefore, are the two states which constitute active and passive hæmorrhage." 162.

Dr. Parry regrets, that he has not seen a sufficient number of cases of sea scurvy, to enable him to decide whether, at a certain period of that disease, there be not excessive momentum that may ultimately produce those extravasations and passive hæmorrhages so characteristic of the disease. We have had extensive opportunities of seeing scurvy, and Dr. Parry's reflections on the subject have recalled to our minds many circumstances favourable to his ideas. We always observed, that the first symptoms of sea scurvy broke out among those who were addicted to drunkenness, laziness, and gluttony, all which undoubtedly tend to produce general or local plethora, with consequent hæmorrhagic discharges. We observed, on the other hand, that after great exertions of body and anxiety of mind, any sudden disappointment, as the escape of a prize, the discomfiture of an attack, or the like, gave origin to a formidable list of scorbutics, which extended even to those of active, sober, and cleanly habits. This we conceive, was owing to the sudden atony of the *extreme vessels* invariably following the application of the *depressing passions* to minds and bodies bordering on exhaustion from previous fatigue, and very fairly accounting for the hæmorrhages and extravasation in question.

The petechial spots in typhus itself are usually the result of undue action of the heart upon skins suffering an accumulation of heat from various causes, as alcohol and other stimulating ingesta, and seldom appear where the treatment goes to regulate the balance of the circulation,

The varicose state of the saphena, giving origin to hæmorrhage, is often certainly the consequence of mechanical obstruction to the return of the blood ; but in many other cases, a diseased state of the coats of the vein is more probably the cause of the morbid dilatation, and of the hæmorrhage.

On the subject of hæmorrhoidal discharges, Dr. P. declines speaking, but refers to the illustrations of Mr. Abernethy. P. 166.

*Final Causes of Inflammation, Dropsy, and Hæmorrhage.*

"In the preceding pages, an attempt has been made to shew a coincidence of action or affection between imflammation, dropsy, and hæmorrhage, inasmuch as each of them is the consequence of excessive momentum of blood, whether relative or absolute."

These actions or affections *of the part* often remove the local malady, which in its turn frequently has a tendency to relieve the general momentum. Blood being the great stimulus to the heart's action, a certain fulness of the vessels, proportioned to the varying state of the system, is necessary for a due action of that organ. Thus excessive quantum increases, defective diminishes the action of the heart, as is evinced by the pulse in arterial and venous plethora following full living, "and the immediate change of that state by blood-letting." 169.

"These circumstances," says Dr. P. "render it probable, that one of the ends to be answered, in such cases, by the supervention of local inflammation, is, in various ways, to evacuate and soothe the constitution, which was before unduly stimulated by excessive vascular fulness." *Ib.*

In this way we partly conceive the salutary influence of gout in constitutions subject to excessive sanguineous momentum, and how other local affections supply the place of gout, as erysipelas, and various cutaneous affections. So also, hæmorrhages and hæmorrhoidal discharges prove vicarious of the same disease; thus our author has seen a patient accustomed to vernal gout, and missing the annual fit, have *erysipelas;* the next spring, *hæmorrhoids;* the following spring, a *fever*, cured by blood-letting; each with equal relief to the constitutional symptoms. The same effect precisely (in his experience) has been produced by anasarcous swellings of the lower extremities occurring at the gouty seasons. These circumstances then, shew, that constitutional errors of circulation are alleviated by local inflammation, hæmorrhage, and dropsy. So also, increased action of the heart is relieved by dropsical effusions. Our author has seen a hectic pulse of 130 reduced in a few hours to 60, by the supervention of violent anasarca in the lower extremities.

"I have, indeed, so often known constitutional maladies suspended, and life evidently lengthened and rendered more comfortable, by the coming on of various dropsical effusions; and, on the contrary, so many persons suffer aggravations of disease, or even death, very shortly after the spontaneous disappearance of dropsy, that I cannot avoid considering that effusion as a salutary process, rather than as an actual disease." 171.

These pothological points are certainly of the utmost importance, both in a speculative and practical view, inasmuch as they direct or sanction modes of treatment so active as to be either essentially beneficial on the one hand, or highly injurious on the other.

The coincidence of hæmorrhage with dropsy is not frequent; but Dr. P. has seen a long-continued and large hæmorrhage from the lungs, accompanied with hydrothorax, anasarca, and ascites, with a pulse at 136, all relieved together, and the patient restored to health as soon as, by digitalis, the pulse was reduced to 40 in the minute. Although dropsy often appears a consequence of hæmorrhage, Dr. P. thinks that it is rather from the *cessation* of hæmorrhage. Where the *latter* is accidental, but in some degree habitual, the dropsy arises either from a similar cessation, or from too sudden nutrition, both of which produce excessive plethora, often resulting from the means employed to arrest the disease and restore the strength!

*Simple Excessive Determination, or Fulness of Blood.*

This is a very long and a very important section in our excellent author's work, and must receive proportionate attention.

Every accurate practitioner must have observed, that long after the subsidence of *actual* inflammation, gout, &c. in parts, an increased momentum of blood, accompanied by pain, swelling, or serous effusions, remained. Pains of various other parts appear to originate in this increased momentum, producing excessive impulse on them when in a state of susceptibility; and hence idiopathic pain in the animal frame can only be accounted for in this way (independent of "*inexplicable nervous sympathy*") "since pressure, bruising, tearing, cutting, stretching, suction, and probably all chemical operations, are mere modifications of that power." 178. One principal, and almost only objection to the doctrines of Dr. Parry, is a total neglect of the *nervous system*.

> "He utterly, and for ever, disclaims all reliance on the *neurological* systems of pathology hitherto extant. He considers them as founded on principles which are either visionary or inapplicable, and which lead to practices tending equally to debase the moral character of mankind, to produce or perpetuate disease, and to discredit the medical profession." Preface, v.

This is too severe, and perhaps unjust. The nervous system must not be overlooked in pathology; for we are quite convinced, from attentive observation and reflection, that whether primarily or secondarily affected in disease, it plays as important a part as the vascular system; in short, that the *two systems* are so mutually dependent on, and connected with each other, that our views must be constantly directed to both, if we wish to practise with discrimination and success. But to return to our author.

> "Pain itself has a tendency to diminish the action of the heart, and therefore that increased momentum or fulness of blood which produced it." 178.

Does not this very sentence of Dr. Parry's admit the mutual connexion we have been insisting on? Dr. Parry justly remarks, that we often see swelling, increased heat or redness, and occasionally all three, without actual inflammation, though these are the common characteristics laid down by Nosologists. Since, however, the two states vibrate backwards and forwards into each other, so that what is one day a *determination* to a part, will the next be *inflammation*, there is evidence of one common condition in the two cases.

These determinations produce different effects in different structures. In the serous and cellular textures, inflammation or effusion is very generally the result. Effusion, in some places, assumes peculiar appearances; viz. in the uvula, a semi-transparent elongation and swelling, &c. when the increased determination is to the skin, heat, redness, and perspiration follow. Although a determination of blood to the nose from drink is frequently *coexistent*, Dr. P. thinks it not at all *connected* with hepatic affection, as suspected by Darwin and others. Dr. Parry here alludes to various anomalous, and indeed unaccountable pains and swellings, short of inflammation, which affect different parts of the human frame. Among these, we may instance that tenderness of the soles of the feet during the intervals of gout; those pains of the hypochondria in nervous females; the pain and soreness of the head in the same description of people, which Dr. Parry, perhaps with much truth, asserts to be peculiarly difficult of relief "under the false theory which dictates the usual treatment." 185. Another example is furnished by the rather sudden and painful swelling of one or both mammæ, alternating frequently with disorders of the head, and unconnected with that uneasiness or tumefaction preceding the menstrual periods. Our author has often seen "enlargements of the uterus with a sense of weight and bearing down, and sometimes with various discharges," yet unattended with fever, or any evidence of local inflammation, frequently disappear, either spontaneously, or under medical treatment. The knowledge of such circumstances ought to make us guarded in our prognosis on such occasions, since it is generally of an unfavourable kind. The prostate and other glands often undergo enlargement without inflammation or scirrhosity. The most remarkable disposition to enlargement without inflammation is evinced in the thyroid gland, constituting goitre or bronchocele.

"I have so often seen this swelling follow diseases of the heart, and other maladies, more especially those called nervous, such as epilepsy, &c. in which the blood is propelled, with excessive momentum, to the vessels of the head, and yet at the same time have observed such sudden augmentations and diminutions of the swelling, that I have suspected the gland itself to be intended as a diverticulum for blood disposed to flow with too great force to that important organ, the brain." 188.

Whether or not it be an accidental concatenation we know not; but in two decided cases of cardiac enlargement, now under our care, the thyroid gland is considerably enlarged. The liver and spleen are well known to suffer enormous turgescence, especially during the cold fits of ague, without inflammation, or any bad consequence. The vascular system of muscular parts also exhibits, on many occasions, these signs of turgescence; as, for instance, in the lower extremities previous to gouty paroxysm, &c. Speaking of those palpitations of the heart which so frequently affect females, when either chlorotic or labouring under strong nervous complaints, and which are excited by any muscular exertion, Dr. P. observes, that "the beating of the heart is usually felt at such a distance from its natural place, and there is often such a difficulty of lying on either side, that one cannot help concluding the heart to have suffered considerable enlargement."

"In such a case," says he, "I have known absorption of two of the ribs carried to a considerable extent; and pressure with the finger on the heart, through the yielding spot, produced great anxiety, and immediate disposition to syncope. Yet this patient recovered, and now enjoys tolerable health, thirty years after the period of my attendance." 191.

We quote this authentic and extraordinary instance, to shew that cardiac diseases should not be considered in quite so melancholy a point of view as they generally are; because we are convinced, from experience, that much may be done by regimen and medicines, even in the worst cases.

Few textures are more liable to increased determinations than mucous membranes, being exposed from situation to the constant influence of chemical and mechanical causes. Inflammation of these membranes is sufficiently common and sufficiently understood; but medical writers have not noticed, "that a state of excessive determination of blood to this membrane, (that of the nose,) though *without inflammation*, gives rise to some very common and important disorders." 195. Dr. P. has seen many instances of violent coryza without the least evidence of inflammation; sometimes by passing into a hot room—one by the internal use of hyoscyamus.

"In patients who are subject to spasmodic asthma, fits of that disorder often begin with a violent coryza, in which the eyes become red and watery, and all the symptoms of a cold in the head are observable. After a few days, or even hours, these symptoms suffer some degree of alleviation, and the malady proceeds to the bronchia, occasioning all the well-known symptoms of spasmodic asthma. What, then, is this state in the bronchia, but an affection of the mucous membrane of those cells, exactly similar to that which had previously existed in the same membrane in the nose?"

We think this a valuable observation. If it be said that asthma is a spasmodic affection depending on causes acting on the mind, &c. and periodical, Dr. P. answers, that the sense of suffocation in hydrothorax, and certain diseases of the heart, returns also at regular periods; while affections of the mind produce, aggravate, and renew gout, acute rheumatism, hæmorrhage, and various other disorders, to which no one thinks of assigning the name spasmodic. Again, what is termed spasmodic asthma is brought on by almost every thing that increases the action of the heart, and stimulates or fills the vessels of the mucous membrane itself; as intense heat, lightness of the air, exercise, full meals, stimulating drinks, certain effluvia—as those of hay, sealing-wax, ipecacuanha, &c. whilst it is relieved by open bowels, heavy air, cold inhalations, and cooling drinks; diminishing as soon as a mucous secretion, or spitting of blood, relieves the turgescence of the vessels. These facts, Dr. P. thinks, are convincing proofs of such a preternatural fulness of the vessels of the mucous membrane of the bronchia, as impedes free inspiration, and produces all the symptoms of spasmodic asthma. There can be little difficulty in understanding how a vascular fulness of the bronchial mucous membrane should produce dyspnœa by mere mechanical diminution of the tubes and cells, when a similar affection of the same membrane in the nose renders it sometimes absolutely impossible for us to respire through the capacious opening of the nostrils; and hence the absurdity, says Dr. P. of assuming asthma to be a nervous disease, produced by a *spasmodic* constriction of the air tubes.

This discussion on asthma clears the way for a knowledge of various other affections of mucous membranes, hitherto improperly termed spasmodic. Thus those strictures in the urethra, which are frequently influenced by mental causes, are probably owing to a turgescence of blood rather than to spasm.

Dyspepsia, Dr. Parry thinks, is a morbid fulness of vessels in the villous coat of the stomach, for the following reasons:—1st. Its symptoms are those of increased sensibility; (which is usually attended with, or produced by, vascular fulness) thus this organ suffers from a quantity of food that in health would produce no uneasiness. 2d. The cardialgia evidently arises from increased turgescence of blood. But here we would ask, how is it, that carbonate of ammonia and other stimulants are the surest means of relief in such cases? 3dly. When vomiting occurs in dyspepsia, when the stomach is void of food, the ejected fluid is merely an unusual quantity of the natural mucous secretion. 4thly. All the symptoms of dyspepsia, as flatus, heartburn,

&c. exist in the greatest degree, in those cases which are followed by vomiting of blood, and are relieved by that discharge till a similar congestion of blood again takes place in the vessels of the stomach. 5thly. As females with obstructed menses are peculiarly liable to this state of dyspepsia, with bloody vomiting, so those who are regular in their catamenial discharges, suffer much less from dyspeptic symptoms *during* those periods, but relapse again in the intervals. A greater degree of this excessive determination then, would probably bring the malady within the limits of inflammation; instances of which, the author thinks he has seen. If, then, idiopathic dyspepsia be excessive vascular fulness of the villous coat of the stomach—if this may be produced by mental affections, and occasionally run within the limits of inflammation, we may conceive the process by which mental affections may be followed by scirrhosity, and subsequent ulceration; also, why the same may be produced by spirituous liquors, and mechanical injury. This discussion on dyspepsia may throw some light on the formation of strictures in the intestines, and also in the œsophagus. In common diarrhœa, an excessive determination of blood to the villous coat of the intestines probably takes place for the purpose of expelling the offending matter.

"I have," says Dr. P. "seen vehement evacuations of this kind continued, by strong purges, for months, and even years; and yet, after death, dissection shewed the entire villous coat of the greater part of the intestinal canal still preternaturally turgid with blood." 210.

We do not doubt it:—the "strong purges" were sufficient to keep up a very constant determination to the bowels; but might not the plethora have been diverted to the skin in such cases, by diaphoretics? or, obviated by abstemiousness and venæsection? This is corroborated by Dr. Parry himself.

"In a boy, seven years of age, there were such motions as I have described (copious and loose) with fever and almost total insensibility. He had, in vain, for sixteen days, tried all the most approved *aperients*, when *six ounces of blood* from the temporal artery, and repeated next day, immediately brought the stools to their natural state, and the patient to convalescence." 211.

Dr. P. gives us some interesting remarks on those pulsations which we sometimes feel in the abdomen, occassioning apprehensions of aneurism. In all persons not very fat, the pulse of the aorta can easily be felt, while lying on the back, if strong pressure be made a little to the left of the median line, about half way between the navel and scrobiculus cordis; in some instances, the pulsation is painfully perceived by the patient himself. In many cases of this kind, particularly in nervous patients, this sense of pulsation is merely the effect of preternatural action of the heart; in others, of the pressure of some hard substance on the descending aorta, determining a disproportionate quantity of blood to the head, "and giving to the hand placed on the abdomen, and sometimes even to the eye, the appearance of a beating so near the surface, as to lead inexperienced observers to conclude that the aorta is morbidly dilated." We are not ashamed to say, that we were once deceived in this way, and our prognosis turning out so wrong, has made us extremely careful since, in ascertaining the true state of the parts. The most common causes are collections of fæces in the colon, requiring repeated and active purgatives, which must bring away almost incredible discharges of stercoraceous matter before the aortal pulsation subsides.

In many cases of dyspepsia, the affection of the stomach is only secondary, while the primary disorder exists in the colon; the villous coat of which seems to be affected with morbid sensibility, unspeakable uneasiness, burning heat, and all those other circumstances which have been described as occurring in the stomach. This state frequently runs into inflammation, and lays the origin of strictures in that intestine.

Fluor albus and gleet are brought forward by Dr. P. as also affording examples of simple excessive determination of blood to mucous membranes. To this is attributed a common species of deafness, described by Dr. James Sims, and usually called nervous, depending on obstruction of the Eustachian tube.

> "The patient can hear well when the tube is distended, by strongly blowing, with the nose, mouth, and cheeks closely shut."

Our author has known it removed by the supervention of hepatitis, and of hemiplegia; returning as these diminished; also entirely to cease, in two instances, forty-eight hours before death; and thirdly, completely cured for more than a year of the remainder of life, by an accidental hæmorrhage from the humeral artery.

The following are instances of increased determinations of blood producing secretions or excretions, morbid or natural. 1st. Spontaneous inordinate ptyalism is often produced by the influence of the mind, for it is frequently the result of an excessive attention to the discharge, or constant attempts to eject it, under the erroneous impression that it is excrementitious. Diabetes will probably be admitted as the effect of an excessive determination of blood to the renal arteries, since the secretion of urine is preternaturally increased; and dissection shews, that the kidneys are preternaturally vascular.

The turgescence of the genital organs in man and in animals is adduced by our author as an illustration of salutary, or at least natural determination of blood to certain parts, intended to be followed by increased secretion or excretion. So are the menstrual periods in women, which are generally preceded by pains in the loins, owing, in a great degree, to turgescence of the hypogastric arteries, and which disappear when the natural discharge is fully established.* The progress of sanguineous momentum is beautifully illustrated by the phænomena of ephemeral fever. Here, at the commencement, the skin and extremities are cold, pale, shrunk, and in some degree benumbed, while the pulse is weak and soft. By degrees, as the action of the heart increases, the impetus of the blood is augmented, first to the head, and then to the trunk. At length, the extremest parts feel the influence of the vital current, and then perspiration breaks out as after violent exercise, diminishing the impetus and bringing relief to all its effects.

---

* In a former note we alluded to the power which the heart possesses of *forcible* dilatation, as well as contraction. The same power may be extended to the vessels also. If, through the influence of the mind and the nerves, for instance, the vessels of the genital organs exert their dilative powers, an increased afflux of blood must necessarily rush to those parts, although it is generally, and we think falsely, considered a *determination* of blood by some power *a tergo*. But, as we have often said, the heart can have no elective power to distribute more blood to one part of the system than to another—it must all depend on the state of the vessels themselves. The vessels of the cheeks, in mental emotions, exemplify this principle; and so do the vessels of any part of the surface if chafed or heated. Their dilative or erectile powers are called into action immediately, and they swell with blood independent of any alteration in the action of the heart or trunks leading to the parts. The above states we would consider active or *erectile* states of dilatation, in contra-distinction to those *passive* dilatations resulting from debility of the vessels of the part. Here they are, in fact, dilated for want of their due proportion of tonic power, as where a part is bruised, burnt, or otherwise debilitated. There can be no doubt, also, that excessive action of the heart, by producing an increased impetus or momentum throughout the whole vascular system, must very frequently cause disorder of particular parts, which may have been previously predisposed to disease, and thus induce all the phenomena of "local determination," as it is called. Keeping this *modus operandi* in view, we think Dr. Parry's facts are exceedingly important, and the result of most accurate observation.—*Rev.*

The same process frequently occurs to one part, or certain parts only, of the body, as the arm or both legs, which go through regular fits of what may be termed ague, beginning with preternatural coldness, and proceeding through excessive heat to termination by sweating. Of this progress of increased impetus, there is, in many women, an exemplification during menstruation.

"One or two days previously to that period, they suffer a violent pain and weight in the head, accompanied with flushing and heat of the forehead and cheeks, occasionally with sickness. In this state the pulsation of the carotids is strong; the feet are often cold, and the general symptoms are those of slight sick head-ache. In the course of one or two days, the head-ache becoming better, the back and loins begin to suffer aching pain; and this is a prelude to the natural discharge, which in a few hours appears; and then, the feet having previously become warm, all the uneasy feelings soon vanish." 223.

Nervous women often suffer sudden determinations to the skin of the face, and sometimes the greater part of the body. It is attended with a flushing and great heat of the skin, and in an instant succeeded by sweating; after which, the skin becomes cold, and the action of the heart is diminished, often in an undue degree. In the latter case, some faintness ensues. All the steps of the process are performed in a very rapid manner, and are called by the vulgar, in the author's part of the country, the hot blooms.

Dr. Parry hazards a conjecture, that fungus hæmatodes itself is occasioned, or at least accompanied, by an increased momentum of blood determining its specific nature.

We shall pass entirely over two sections in our author's work—one on the structure and functions of the Nervous System; the other on the Mental Faculties. In the *first* are many accurate and ingenious anatomical remarks, though we think he has eulogized rather too highly, Messrs. Gall and Spurzheim. In the second, there is much acute reasoning, and much good sense. The following paragraph we extract:—

"Although, however, sensations, and thence the ideas which grow out of them, are the materials from which we think, judge, and act, it is evident that they would little avail us, were there not *superadded* that capacity of *employing* them, which constitutes the faculties, by the degree or nature of which man is chiefly distinguished from other animals." 278.

The Nervous Constitution. The comprehensive term "*nervous*" has been applied to almost all inordinate movements of the parts concerned in the functions both of the animal and organic life, and to nearly all morbid states of sensation. This constitution shews itself by an extraordinary degree of sensibility and irritability; in consequence of which, certain impressions that, in a well adjusted constitution, are either indifferent or pleasurable, produce pain, inordinate actions, or both. Those who, from the habit of self-indulgence, the vicious compliance of parents, the indolence and luxury of wealth, or sedentary occupations, are exempted from the irritations and pains of body and mind, which Providence has made essential to our well-being in this probationary state, are the certain victims to this Proteian malady! The same causes produce nearly the same effects on various animals under the subjection of man.

As simple sensations become less acute by frequent excitement, we may readily conceive, that the causes which predispose to the nervous temperament, act immediately on the brain itself, as the ultimate organ of sensibility. It is true, that many parts of the organic life, as the heart, alimentary canal, &c. become inordinately irritable in nervous constitutions, but even here, we find, that the influence of mental impressions, acting through the brain on these various parts, is greatly lessened by the mere repetition of the irritation; so that a boy who starts with terror at the report of a gun, will, after having been a few weeks in the naval service, himself fire a cannon without the smallest trepidation.

Although original conformation may predispose individuals to be more or less easily acted on by the various causes of excitement, yet it is questionable, whether this state of morbid excitability, from long exemption from the causes of excitement, would reach that extent of disease which we see in the nervous temperament without the co-operation of some other cause.

"I think," says our author, "that such a concurrent cause does actually exist; and that this cause is excessive impetus of blood, acting on the medullary substance of the brain, or some other part of the encephalon." 292.

It must also be kept in mind, that increased impetus of blood not only excites actual disorder, but disposes the brain to be more easily acted on by other causes of irritation than if that excessive impetus of blood did not exist. And as this excessive determination to the brain does not, for the most part, produce its morbid effects until it has continued for some time, it is reasonable, thinks our author, to suppose, that this impetus is itself capable of aggravating or causing that inherent state of the brain termed original predisposition to excessive excitability. 294.

"If this be true, we may, perhaps, profitably carry our investigation one step farther, and inquire, whether the whole of this predisposition may not, in all cases, be formed through the medium of the *sanguiferous system;* so that the exemption from impressions, &c. above stated, as a cause of such diseases, may itself produce its primary influence on that system, while the brain may suffer only secondarily; but in its turn, re-act on the sanguiferous system. Thus, for the sake of illustration, let us suppose, that, from indolence or other causes, the heart has acquired an excessive morbid irritability. In this case, any impression communicated to it from the brain, may excite in it inordinate action, which determining the blood with excessive violence to the brain, may cause it to re-act on various other parts, and thus produce the phænomena of nervous disease." 295.

Dr. Parry, so long back as the year 1788, attempted to prove that nearly all the modifications of nervous disorders originate in excessive momentum of blood in the vessels of the brain.* He there shewed that excessive sensibility in regard to external impressions, head-ache, vertigo, spasmodic dyspnœa, hiccup, general convulsions, and delirium, might be, for a while, wholly removed, or greatly mitigated, by compression of the carotid arteries. He asserts, that subsequent experience has added irresistible force to the above conclusion. Our author illustrates his pathological views by numerous apposite facts and observations. Thus the pulsation of the carotids in nervous persons, and in the nervous states of those persons, is preternaturally strong. The head is usually much hotter, and the face redder than in a state of health. *Insomnium* is brought on by excessive bodily or mental exertion, by anxiety, late hours, hot rooms, spectacles that strongly arrest the attention, frequent succession of objects which dazzle the eyes, &c. It is usually accompanied with cold feet, præternatural action of the heart, and throbbing of the carotids.

"Under these circumstances, sleep has been, on numerous occasions, induced by lying on one side, and making with the thumb a firm compression on one carotid artery."

Those strange noises which nervous persons are accustomed to hear, are supposed by our author to be occasioned by "the rush of arterial blood through some part of the vascular system of the ear," as they are apt to be produced by whatever increases the action of the heart, as hot rooms, late hours, long watching, strong drink, violent muscular exertion, excessive mental attention, &c. and are diminished

---

* Memoirs of the Medical Society of London, vol. iii. p. 77.

by all those causes which have a contrary tendency. When the rushing sound is waving or alternate, which it often is, it is exactly synchronous with the systoles of the heart. Our author has always been able to remove it entirely, *pro tempore*, and always to alleviate it, by compressing the carotid of that side. Nervous head-aches, whether affecting the external or internal part of the head, are owing to corresponding conditions of the circulation in the *external* or *internal* carotid. That which occurs from dyspepsia, or disordered peristaltic motion of the intestinal canal, is usually of the *first* kind, often extending itself to the muscles of the neck, accompanied with flushing of the face, strong pulsation of the carotids and their external branches. It may be relieved by strong pressure on the common trunks, in consequence of which, the peristaltic movement of the alimentary canal is often increased, the heat of the head is diminished, and the feet, if previously cold, become warm. The SICK HEAD-ACHE exemplifies that which arises from excessive determination of blood to the branches of the internal carotid. It is generally attributed to derangement in the liver or alimentary canal; but our author conceives that the state of the stomach is the *effect* not the *cause* of the malady in the head, which it *never precedes*, just as sickness and vomiting are the consequences and not the cause of the affection of the head, produced by a blow on the cranium.

"Accordingly, the sick head-ache may be cured or relieved by spontaneous bleeding from the nose, or other similar remedies applied to the head; but is not alleviated by purging, and is always aggravated by the stimulants which relieve dyspepsia."

Vertigo and epilepsy are explained on the same principles. The latter, says Dr. Parry, "whatever may be its primary causes, usually depends immediately on excessive impetus of blood in the vessels of the brain." Thus the symptoms of epilepsy are more or less of a convulsive, or even strongly contracted state of various muscles—chiefly those of the eyes, face, tongue, neck, throat, upper extremities and respiration, accompanied with loss of sense, and followed by stupor. Those parts are chiefly supplied with nerves from the encephalon, whose functions are greatly disturbed. The suddenness of the attacks, and the perfect intervals which exist between them, imply the operation of a fluctuating cause, which we cannot conceive to be any other than one of those sudden changes in the balance of the circulation which we continually see occurring in the sanguiferous system. When it occurs at an advanced age, it chiefly attacks those who have long been constitutionally nervous, or who have lost the accustomed excessive sanguineous determinations of gout, hæmorrhages from the nose, hæmorrhoids, ulcers, eruptions, &c. and in all these cases, the pulse in the carotid arteries is habitually stronger than natural. Lastly, it often terminates in, or is exchanged for, sanguineous or serous extravasation in the brain, and consequent hemiplegia or apoplexy. Epileptic attacks are more or less prevented by whatever habitually diminishes the excessive action of the heart, or lessens the flow of blood to the head.

"Thus it is often superseded by gout. In more than one instance, the paroxysm has been removed by the affusion of cold water; and in others, by compressing the carotid arteries."

Dr. Parry very justly observes, that, where epilepsy arises from exostoses or other local diseases in the cranium, even here, the disease still attacks only in paroxysms; hence, the *local* disorders act merely as causes of predisposition, requiring for their production of the fit the coincidence of those causes which manifestly operate by increasing the impetus of blood to the head. But it may be doubted, whether these local derangements of structure also are not attributable to the same vascular impetus.

Convulsions are next adduced by Dr. Parry, as resulting from the same cause; and in fact, as a modification of epilepsy. Chorea is also considered by our author to be frequently dependent on the same cause, as are hysteria and hypochondriasis. At page 346, Dr. Parry gives an explanation of what he means by particular determinations. He admits, (and we fully coincide with him) the existence of excessive *local* momentum without excessive *general* momentnm.

"Thus I have many times known the pulse in the *temporal* artery so weak that blood would not flow from it, however well it was punctured; and other instances, in which it was too weak to be felt; and yet, in all, the pulse in the carotid artery has been extremely strong, and there has been the most decisive evidence of preternatural *impulse in the brain*. If, therefore, such a difference of impulse can exist in two sets of vessels derived from the same trunk, and so near to each other, we may readily conceive the internal branches and the capillaries arising from this artery, to be on other occasions, sufficiently full to produce all the symptoms, *without any increase* of fullness in the *trunk* of the carotid."

Hydrocephalus is another disease brought forward by our author, as exhibiting proofs of increased impetus to the brain; and also apoplexy. He thus concludes:—

"From the foregoing relation of facts, I think it clearly appears, first, that a large proportion of nervous affections originates in a disordered state of the circulation with regard to the brain; just as inflammation, hæmorrhage, dropsy, and the various other maladies, which I have specified, arise from similar states of the circulation in other parts; and secondly, that this state is either relative, or absolute excess of momentum, impetus, or determination of blood, in some portion of the arterial system, of the part affected." 358.

Our own observations have long led us to conclusions nearly similar.

Speaking of tic douloureux, Dr. P. observes—

"All the circumstances induce me to attribute this pain to increased vascularity or determination of blood (perhaps amounting to inflammation) to the neurilema, or vascular membranous envelope of those nerves."

He thinks that the operation of cutting the nerve (performed by Dr. Haighton) was rather the division of the arterial branch supplying the affected ramification of the trigeminus nerve, than the division of that ramification itself.

A very interesting section follows, on "one common origin of diseases;" from which we shall extract as much as our narrowing limits will allow. As it is acknowledged that one family is more liable than another to scrofula, another to gout, a third to eruptive complaints, a fourth to mania, &c. so in different individuals of the *same* family there is a resemblance of modification in the several affections, proving them to be only varieties of the same common stock. Thus, in regard to the *head*, he has known one person maniacal; a paternal cousin hæmorrhagic and epileptic; and almost all his children subject either to epilepsy, head-ache, epistaxis, or hydrocephalus.* In another family, the mother was *epileptic;* a son laboured under excruciating *head-aches;* a daughter died of *hydrocephalus.* Of two sisters, one had *eruptions* on the face; the other, flushing *heat of head,* with nervous affections. Of two other sisters, one died (adult) of *hydrocephalus;* the other had *head-ache, hysteria,* and *erysipelas.* In another family, a female had *epistaxis;* her sister had *nervous symptoms;* and two brothers were *maniacal.*

The extension of these diseases in different forms, and therefore under different names, to different parts nearly at the same time, is very interestingly illustrated by Dr. Parry.

---

* Our readers will see the coincidence between Drs. Parry and Prichard on these subjects. Indeed there are few *good* pathological doctrines of the present day which may not be found in Dr. P's work.

Many diseases appear to extend, by being joint affections of different or even remote branches of the same arterial trunks. Thus Dr. P. saw a man who, with violent rheumatic inflammation in the right shoulder, had a pulse in that wrist considerably fuller than in the other; that arm and hand were also hotter, and disposed to sweat, while the other was quite the reverse. When determination of blood takes place to the *bowels* in diarrhœas, &c. the muscles of the *thighs and legs* are often affected with pains, cramps, &c. and the feet are burning hot.

"During a gouty diathesis, a brisk purgative will often produce acutely inflammatory gout in the knees or feet." 372.

The purgative produces, we suppose, increased determination in the mesenteric and hypogastric arteries, and ultimately in the arteries supplying the lower extremities. Determinations of blood to the uterus, on the same principle, produce pains in the loins, groins, and down the thighs. These examples may suffice. Several curious examples of the "relation of diseases by remote changes" are adduced, of which we shall select a few.

Examples are very common where the same patients shall have, at different periods, hæmorrhoids, head-ache, vertigo, erysipelas, gout. Others, where a constitutional tendency to these will end in epilepsy, hemiplegia, or apoplexy. In one patient the succession was, gout, mania, and at last fatal epilepsy. In several instances, fits of epilepsy superseded gout. In a gentleman, an intemperate liver, gout, to which he was long subject, ceased, after an abscess with great discharge from the neck. In a gentleman, the cessation of gout was followed by cough, difficulty of breathing, anasarca, defective urine. These being cured, he was seized with a loss of sense, unattended by convulsions. He recovered, and was affected with apthæ all over his mouth and throat. No sooner was he recovered from this, than the original disease, gout, returned, and became regular. Another patient had atonic gout, with quick pulse; defective, high-coloured urine; legs and thighs enormously swelled; and such a difficulty of breathing, apparently from hydrothorax, that for forty nights he had not even attempted to go into a bed; yet in a few days, by appropriate remedies, he lost every symptom of disease. And now a spontaneous and acute fit of gout came on, which terminated in perfect health. A lady, habitually subject to diarrhœa, fell into a state of costivenesss. Some months afterwards, she was suddenly seized with giddiness and head-ache, accompanied with fever, followed by almost apoplectic insensibility, great heat of the head and face, with other symptoms of erysipelas. These disappeared after three or four days, and she returned to her former state of costiveness. Five months afterwards, pain and giddiness in the head, with erysipelas on the left side of the face, again occurred. And now there came on, by degrees, hemiplegia of the left side, together with loss of speech.

Conversion of Diseases. This Section is so extremely interesting, that we shall endeavour to condense as much as possible of it for our readers.—1. In a gentleman, the pain of a node on the shin alternated with vertigo and a sense of numbness in the head. 2. A head-ache, of some years' duration, subsided, and was followed by a cough, and incessant wasting hectic fever. After the man had been long confined to bed, and death was every day expected, the head-ache began slightly to return; and as it became established, the cough and fever subsided. 3. During vertiginous and other distressing complaints of the head, carditis twice or thrice occurred and suspended them; they immediately returned as the carditis abated. 4. Slight paralysis of the hands alternated with spasmodic asthma. 5. Vertigo and hæmorrhoids very commonly alternate. 6. In a gentleman, vertigo and head-ache were constantly relieved by œdematous swellings in the legs and feet. 7. In a lady, mania, ending in suicide, alternated with œdema of the ankles. 8. Fits of spasmodic asthma alternated with gout. 9. A gentleman lost epilepsy on being attacked with gout, a paroxysm of which was immediately followed by a sudden attack of asthma, which proved fatal in twenty minutes!

10. "On the other hand, various diseases of the head, as head-ache, vertigo, depression of spirits, mania, epilepsy, and apoplexy, in many instances, immediately or soon succeed the recession of inflammatory gout from the extremities." 382.

Let this be a caution to Dr. Kinglake, who endeavours to brow-beat every proof of the fatal effects of his favourite remedy. Vide *Med. and Phys. Journ. passim.*

11. Recession of gout in a clergyman was followed by slight hæmorrhage from the rectum; which ceasing, fatal epilepsy supervened. 12. Epilepsy was superseded by pneumonia. 13. Vomiting of blood and mania alternated. 14. Bronchocele disappeared during hepatic inflammation. 15. Long-continued cough, hectic fever, emaciation, and night-sweats, ceased spontaneously on the breaking out of an ulcer on the scapula. 16. Orthopnœa and cough, of many years standing, suffered a sudden and violent aggravation. Œdematous swellings of the lower extremities and scrotum, with scanty urine, removed the orthopnœa entirely. After some weeks, the urinary secretion returned to the natural quantity, and the œdema vanished. Mental alienation now gradually succeeded, and, in a few months, gave way in its turn to asthma, which continued during the remainder of the gentleman's life. 17. The alternation of cutaneous eruptions with dyspepsia is well known. 18. That of the same disorders, with asthma and other forms of dyspnœa, has, in Dr. P's experience, been full as frequent, and much more important.

19 "I have often," says Dr. P. "seen various thoracic affections, as pulmonary consumption, asthma, carditis, or hydrothorax, arise from the spontaneous or artificial cure of ulcers, perpetual blisters, or fistulæ." 386.

It has lately been the fashion to ridicule these things; but we would seriously recommend the inexperienced and unobservant practitioner to ponder on the foregoing deduction from long experience.

20. Gout and erysipelas alternate. 21. An instance, where long-continued symptoms of apparently pulmonary hectic were entirely removed by a frequent and copious nasal hæmorrhage, which disease itself ultimately proved fatal. 22. Cough and bloody expectoration ceased on the supervention of œdema in the lower extremities; but returned, in a fatal degree, when the œdema vanished. 23. Many instances where extensive œdema ceased from violent spontaneous hæmorrhage. 24. Chronic bronchitis, or asthma humidum, is frequently relieved by œdema in the lower extremities. 25. A gentleman spat blood copiously every day for twenty years, during which he abstained from animal food and strong drink. Attempting to return to animal food by slow degrees, he had, in one year, four attacks of inflammatory fever. These were succeeded by vehement palpitation of the heart, which frequently returned during several years. They ceased on the supervention of cough and copious expectoration of mucus. After some years, the cough and expectoration disappeared, and were succeeded by dyspepsia and the original palpitation. These gave way to remedies, but were immediately followed by hæmoptoe. From this period, during the remainder of his life, which was extended to 80, the three states, of mucous expectoration, hæmoptoe, and palpitation, alternated with each other; but no two of them ever existed together. 26. Habitual cough, dyspnœa, expectoration, and deafness, were nearly cured by hemiplegia, but returned as the hemiplegia was relieved. 27. Habitual cough and expectoration were suspended by rheumatic pain on one side of the head, and returned as the latter disappeared. 28. Two instances occurred where the cessation of pleurisy was followed by peritonitis. 29. In several cases, pleurisy was converted into fatal inflammation of the cerebral coverings. 30. A gentleman was, for many years, so harassed by difficulty of breathing, cough, and copious expectoration, that he could scarcely ever lie down in bed. On his being seized with a painful erythema on the scalp, followed by deep sloughs, and accompanied with fever, the pulmonary symptoms entirely ceased. As the sloughs grew well, mania supervened; but, after a short time, was cured by low diet and depletion. From this time he remained free from complaint. 31. The disappearance of scarlatina is well known to be followed occasionally by bloody urine, arthritis, œdema of the extremities, ascites. To these may be added convulsions and epilepsy, both of which Dr. P. has seen. 32. A lady, for several years, had itching and smarting of the anus, with slight serous or mucous discharge. These disappeared, and were succeeded by violent catarrhal affection extending to the Eustachian tube; and when worst, to the bronchia, producing great stricture without cough. These symptoms continued long, and on the return of the affection of the anus disappeared. 33. A gentleman, who had long laboured under vomiting, was no sooner cured of it, than he became anasarcous. A spontaneous purging coming on, the anasarca disappeared. 34. Hæmorrhoids and rheumatism alternated.

35. "A gentleman had the following succession of maladies: Gout, often alternating with enteritis, followed by apoplexy and hemiplegia. The latter complaints were relieved. Then occurred enteritis, and in its place, an almost total want of the secretion of urine, without fever. This last symptom was succeeded by gout, which gave place to fever, attended with erysipelatous inflammation of the stomach, and fatal sanguineous vomiting, during which the urine was restored to its natural colour and quality." 392.

36. A girl, aged eight, had long a mucous discharge from the vagina. This ceased, and the eyelids became inflamed. The latter malady disappearing, the former returned. 37. A lady, who, for many years, had been afflicted with cough and difficulty of breathing, was immediately and permanently cured by a large hæmorrhage from the humeral artery. 38. An old man, who had lived freely, had a chronic inflammation in one leg, accompanied with œdema. Both were greatly relieved by the application of a tight bandage. In a few days he was seized, for the first time, with epilepsy. 39.

A young chlorotic lady had extensive œdema of the lower extremities. This was removed by bandages, when she was immediately seized with a painful affection of the right side of the head, which was always much relieved by a flow of tears from the eye of that side.

40 "A girl, seventeen years old, had a chronic ulceration of the foot. No sooner was this cured, when she was seized with a disease and enlargement of the heart, which proved fatal."

41. In a lady, colica pictonum, with palsy of the hands, not arising from lead, were cured by the Bath waters. Four years afterwards, she had sciatica for five months. Stimulating friction immediately relieved the pain, but, in a few hours afterwards, was followed by a return of the colic, succeeded by palsy of the hands as before. 42. A gentleman had habitual excessive perspiration, which was cured. Immediately he became affected with hydrothorax, anasrca, and ascites; all of which, however, were happily removed by digitalis.

43. "In two cases, which occurred between twenty and thirty years ago, immersion of a gouty foot in cold water, which produced instant relief of the pain, and a proportionate abatement of the inflammation, was, in a few hours, followed by hemiplegia." 396.

Let Dr. Kinglake explain away these facts by a quibbling jargon of unintelligible reasoning! We again refer to his papers in the *Med. and Phys. Journal* for an apology for the harsh expressions here used: but we cannot suppress our indignation when we see human life sported with to support a theory. Dr. K. seems to think, that the generality of practitioners adopt his treatment in gout. We appeal to the knowledge of every individual of the faculty, whether one in fifty of his acquaintances ever dreams of following Dr. Kinglake's plans.

Dr. Parry sums up the effects of *increased* momentum of blood in the following manner, viz.

"First, excessive determination or momentum of blood to the skin, produces—sweating, scarlatina, measles, erythema, erysipelas, and all the forms of eruptive diseases. Secondly, *to mucous membranes*; coryza, catarrh, whooping cough, croup, sore-throat, peripneumonia notha, catarrhus senilis; bronchitis, asthma; apthæ, dyspepsia, diarrhœa; and various other disorders of the villous coat of the alimentary canal; strictures in the urethra, œsophagus, colon, and rectum; gleet; fluor albus; catarrhus vesicæ. Thirdly, *to serous membranes*; phlegmon; pleurisy; pericarditis; peritonitis of different parts, constituting enteritis, puerperal fever, &c. inflammation of the tunica vaginalis testis.—*To synovial membranes*; producing arthritis; together with the effects of these several states, anasarca, hydrothorax, hydropericardium, ascites, hydrocele, effusions into joints, adhesions, anchylosis, &c. &c. Fourthly, *to various other membranes;* of the spinal marrow or nerves, paraplegia, sciatica, tic douloureux, &c. *To the epithelion,* deafness. Fifthly, *to glandular parts;* cynanche parotidœa, or mumps; swelling and other disorders of the thyroid gland, mammæ, testicles, prostate, and various other glandular parts; phthisis pulmonalis; atrophy. Sixthly, *to the head;* head-ache, vertigo, sleeplessness, common nervous affections, mania, delirium, convulsions, hysteria, epilepsy, catalepsy, inflammation of the pia mater, or arachnoides; together with their occasional sequelæ, hemiplegia, apoplexy, hydrocephalus, and other effusions. Seventhly, to other parts in various forms, peripneumony, enlargement of the heart, liver, spleen, kidnies, testicles, and uterus, with or without inflammation; fungus hæmatodes, ophthalmia, cataract, amauroris. Eighthly, *various increased natural discharges,* not already specified; ptyalism, diabetes, lachrymatio. Ninthly, *morbid depositions,* not above arranged; scirrhosites, indurations, ossifications, chalk stone, biliary and renal calculi, and other hard deposits in different parts. Tenthly, *hæmorrhages,* from serous, mucous, or other membranes, or parenchyma; as from the nose, uvula, fauces, lungs, stomach, intestines, kidneys, bladder, uterus, vasa deferentia, skin, liver, &c. To which may be added the various forms of purpura."

Such are the chief examples, according to our author, of diseases in which increased impetus, momentum, or determination of blood, forms one essential link in the series of previous phenomena or causes. In what particular cases they may depend merely on excessive *local* momentum, and in what there is the coincidence of increased action of the heart, it may be difficult to decide. The same symptoms may arise under both states.

When the above list is compared with that of diseases from *deficient* momentum of blood, the contrast would surprise a Brunonian or routine practitioner, where every complaint exhibiting any trait of *debility* is attributed to want of blood—*poorness* of blood, &c. instead of irregular determination and *partial* plethora.

We shall glance hastily at the principal diseases of *defective* determination. It may here be observed, however, that *excessive* determination to *one* part must of necessity produce *diminished* determination to *others;* but the *former* is the most formidable, and eclipses the latter.

1st. As the blood is the material of growth and secretion, we may conclude, that where there is a *partial* defect of these, there is not a sufficient afflux of blood to the part. Thus muscles shrink when not used; the heart becomes flaccid and exhausted, from unknown causes or from obstruction in the coronary arteries. 2d. To the same may be attributed the sinking in of one eye; sudden greyness of the hair, &c. 3d. Dr. P. knew three instances of sudden and entire failure of the pulse in the humeral artery and its branches, while the action of the heart and of the other arteries was perfect. 4th. Defective menstruation. 5th. It is highly probable that costiveness, arising from want of the due peristaltic motion of the alimentary canal, is dependent on the defect of a due momentum of blood in the arterial system of that part. Thus Dr. P. has often known compression of the carotids relieve head-ache and flushing of the face, increase the warmth of the lower extremities, and at the same time produce a glow in the stomach and bowels, and a sensible propulsion forwards of the contents of the intestinal canal. 6th. In mortification from the ligature of arteries. 7th. Syncope from hæmorrhage, or various other causes preventing an adequate quantity of blood flowing to the brain.* 8th. Those gradual hæmorrhages from the nose, stomach, and hæmorrhoidal or uterine vessels, where the patient dies—"with an accumulation of blood about the heart and lungs"—and probably he might have added the *brain*, as the experiments of Dr. Seeds would lead us to expect.

"These," says Dr. P. "are all the cases of disease, whether topical or general, arising from defective determination or supply of blood, that I think it necessary to particularize here." 407.

Far as we have exceeded our boundaries, we must yet spare a few pages for the last Section of this invaluable volume.

*Exemplifications of salutary processes.*—That power of the constitution, termed RE-ACTION, though derided by some of our sciolists in medicine, most obviously shews itself by an unusual redness and glow of warmth which are perceived in parts that have been preternaturally cooled; and evidently arises from an increased determination of blood to the parts, succeeding a deficient afflux of that fluid. In youth and strong health, this reaction produces no inconvenience; but occasionally it proceeds a step farther than is necessary for the well being of the part, and some morbid affection follows. This morbid affection is inflammation, differing in name and symptoms according to the texture of the parts. There is no disease, in which the process of reaction is more apparent than in the common fit of an ague; in this process, one of the most conspicuous circumstances

---

* Dr. Seeds's experiments prove, that there is an accumulation of blood in the brain at these times.

is the occurrence of *shivering* which, as a modification of exercise, our author ingeniously supposes to be an effort of Nature to restore the balance of the circulation and heat to parts in which both were defective.

Dr. Parry considers convulsions themselves as only another modification, and perhaps a greater degree of the same state of tremor, and that they are salutary efforts of Nature to restore the balance of the circulation, in epilepsy and hysteria for example.

"So, under various modifications of nervous diseases, the patient shall sometimes have vehement fits of spasmodic coughing, and at other times, frequent vomitings; one purpose of which several motions is, to drive forwards the blood in the veins, and thus to promote a free and equable circulation of that fluid throughout the system." 416.

This is a very different operation from that of heat, wine, full meals, certain passions, and various other stimuli. *These* indeed cause the heart to produce an inordinate momentum of blood in the several branches of the *arterial* system, and more especially of the head, while the *venous* system is only secondarily and imperfectly acted on. Exercise, on the contrary, by urging forwards the blood in the veins, permits a ready evacuation of arterial blood into those channels; just as opening a vein produces a quicker determination of blood from the neighbouring vessels.

"Under impressions of sorrow, suspense, &c. not only is the patient relieved by tears, which unload certain branches of the carotid artery, but considerable mental ease is obtained by that species of deep inspiration, called sighing, by which the right auricle, and therefore the jugular veins, and the whole nervous system of the brain, are, in an unusual degree, emptied of their blood." 418.

By what catenation such salutary efforts are excited in the system, our author pretends not to explain. The facts themselves are offered as suggestions for the purpose of inducing further enquiry.

An excellent exemplification of reaction is afforded in GOUT. Our author has already considered this malady as evacuating and depleting the system; here he views it in the light also of "a powerful means of restoring the due balance of the circulation, or at least of changing the direction of excessive momentum of blood." He has suggested, that while indolence and habitual indulgence dispose the body to fall into disease, from even a slight plethora, these two habits, growing so much out of the present construction of civilized society, are apt to produce plethora itself. The diseases thus produced, are evidently those of irregular determination of blood, especially to the *head* and *alimentary canal*, parts that almost invariably suffer previously to, or during the intervals of gouty paroxysms. This is evinced on one hand by flatulency, predominant acidity, heart-burn, irregularity of appetite and bowels, and different degrees of sickness: on the other, by listnessness, incapacity of attention, depression of spirits, dreaming sleep, weight or pain in the head, vertigo, &c. Now during this excessive determination of blood to these important organs of the animal frame, there is, often, an unusual degree of coldness in the lower extremities, naturally proceeding from the defective balance of circulation. "Such is the state of circulation in the extremities, which usually precedes, and probably causes, the *reaction* of the constitution," which reaction, in an early period of life, is sometimes a mere aching and preternatural heat of the extremities, and perhaps, occasional cramps; "but at more advanced periods, especially in persons who have been subject to excessive determinations of blood to the head and alimentary canal, producing the symptoms above described, the reaction goes on to the extent of causing gout, erysipelas, anasarca, or other inflammatory affections of the lower extremities." 424. In these paroxysms, the aid of the heart is usually, but not always contributed towards the restoration of the long *defective* determination; and by this process, as by that of ague, the constitution is, for a greater or less length of time, relieved from those disorders, or from that tendency to disorder, under which it had before suffered.

In the first beginning of gout, a very short period seems sufficient to restore the balance of the circulation, and the patient is absolved by one fit of inflammation of thirty-six or forty-eight hours duration in a single joint, which is followed by œdematous swellings, and the speedy recovery of health. But at a more advanced period of life, each attack of gout consists of several distinct inflammations of different parts, occurring in succession, with short intervals, during which, not only those parts of the extremities, which have *not* been affected, still remain preternaturally cold; but even the toes shall be cold, while the instep of the foot suffers burning heat. 427. Thus the disorder proceeds till, if the progress be favourable, a complete restoration of warmth in the extremities ensues. Thus while one final end of gout may be, the evacuation of the habit, and the consequent reduction of a plethora which is relatively excessive, another end is the restoration of the due balance of circulation previously determined in excess towards other and more vital parts. Such are the pathological views of our author on the important subject of gout; and as they are the deductions of experience in a mind of no ordinary power of discrimination, they are of more value than all the chaotic speculations on that disease with which the public have been nauseated, from the *water-works* at Taunton to the *quackeries* of Montpellier; all of which have the same Lethean tendency, in the opinion of more than ourselves.

"If the representation, which has thus been given be just, we can well understand why many local diseases cannot be removed, or even in a certain degree checked, by local remedies, without the hazard of converting a topical into a more general malady, or of causing a constitutional effort on some other part; which part may be more essential to life, than that which the attempt was made to relieve. The same evils may attend the administration of certain internal remedies, the tendency of which is not to cure the constitution, and so remove the necessity of the local disease, but merely to check the present salutary action of the system, and thus to cause only a temporary and delusive supension of present suffering. Such, in the far greater namber of instances, is precisely the action of the *Eau Medicinale* of Husson; the injurious, and even fatal effects of which, local circumstances give me peculiar opportunities of witnessing."

We have now closed the longest analysis that perhaps has ever been given of a Medical Work in any Review. The ideas and the facts of our author were in such a state of concentration, that it required unusual exertions on our part, to exhibit the prominent features of this excellent work in any reasonable compass. We sincerely trust—nay, we confidently hope, that our labours in this article will contribute towards the accomplishment of two objects—the diffusion of the volume already published through the profession at large, and the completion of the work by the author's son—a task which seems to have been prophetically announced by Dr. Parry himself, in the preface to the work before us. If we be successful in these attempts, we shall not have lived in vain—if unsuccessful, we have at least the consolation of having done our duty.

---

P. S. We have just learnt that Dr. Parry has paid the debt of Nature. His works are the best monument of his fame—and the foregoing analysis is the best eulogy which we could pronounce.

